# Melanoma Cell Adhesion Molecule Plays a Pivotal Role in Proliferation, Migration, Tumor Immune Microenvironment, and Immunotherapy in Colorectal Cancer

**DOI:** 10.1002/cam4.70740

**Published:** 2025-03-05

**Authors:** Jingkai Zhou, Jing Liu, Yali Yu, Haihang Nie, Yuntian Hong, Yumei Ning, Chao Yang, Jun Lai, Haizhou Wang, Xuelian Tang, Fan Wang, Qiu Zhao

**Affiliations:** ^1^ Department of Gastroenterology Zhongnan Hospital of Wuhan University Wuhan China; ^2^ Hubei Provincial Clinical Research Center for Intestinal and Colorectal Diseases Wuhan China; ^3^ Hubei Key Laboratory of Intestinal and Colorectal Diseases Zhongnan Hospital of Wuhan University Wuhan China; ^4^ Emergency Medicine Center Zhongnan Hospital of Wuhan University Wuhan China; ^5^ Department of Radiology Xiangyang Central Hospital, Affiliated Hospital of Hubei University of Arts and Science Xiangyang China; ^6^ The Infirmary of Hangzhou Power Supply Company of State Grid, Zhejiang Electric Power Co., Ltd Hangzhou China

**Keywords:** colorectal cancer, EMT, macrophages, MCAM, TIDE

## Abstract

**Introduction:**

MCAM, alternatively referred to as CD146, is an integral membrane glycoprotein belonging to the immunoglobulin superfamily. However, its importance in the tumorigenesis of colorectal cancer is still partially understood. Therefore, this study was designed to investigate the significance of MCAM in colorectal cancer.

**Methods:**

MCAM expression was analyzed by TCGA and GEO databases. qRT‐PCR and IHC analysis were conducted to validate MCAM expression in patient tissues. The tumor‐inhibiting ability of MCAM was further assessed by CCK‐8 assay, colony formation assay, and wound‐healing assay. qRT‐PCR and WB analysis were conducted to evaluate the expression of EMT markers and MMP2/9. qRT‐PCR analysis was utilized to detect the polarization status of macrophages. Kaplan–Meier curve, univariate, and multivariate cox analyses were employed to verify the ability of MCAM in prognosis prediction. TIDE scores were used to assess the impact of MCAM on immunotherapy.

**Results:**

The expression of MCAM was significantly downregulated in CRC, and low MCAM expression revealed poor prognosis in CRC patients. Moreover, MCAM overexpression inhibited the proliferation, migration, and invasive ability of CRC cells. Additionally, MCAM overexpression suppressed N‐cadherin and MMP2/9 expression. Furthermore, MCAM impacted M1 macrophage polarization. MCAM is an independent predictor of CRC patient prognosis through Cox regression analysis. Lastly, TIDE score analysis indicated that elevated expression of MCAM increased immunotherapy efficacy.

**Conclusion:**

The findings of this research suggest that MCAM impacts M1 macrophage polarization and enhances immunotherapy efficacy, underscoring its potential as a therapeutic target for colorectal cancer.

## 1 Introduction

Colorectal cancer (CRC) constitutes approximately 10% of all cancers and cancer‐related deaths diagnosed annually on a global scale [1]. Despite significant advancements in surgical techniques and medical therapies, the cure rates and long‐term survival rates for CRC patients have remained relatively stagnant over the past few decades [2]. The possible reason is that tumor cells interact with tumor microenvironment (TME) components that play key roles in promoting tumorigenesis, progression, and drug resistance by sending external signals (growth factors and cytokines) to the tumors [3]. Therefore, it is imperative to search for an effective biomarker that can not only accurately predict prognosis but also effectively regulate the TME in CRC [4].

Melanoma cell adhesion molecule (MCAM), also known as cluster of differentiation 146 (CD146), is a key protein found on the surface of cells. It belongs to a family of proteins called immunoglobulins (Ig) and was first discovered in melanoma cells. This protein is often associated with a worse outlook for patients [5, 6]. CD146 is a cell adhesion molecule (CAM) that may be activated through homophilic cell–cell or heterophilic interactions with cell–extracellular matrix (ECM) [7]. More and more evidence highlights the significant role of CAMs in various pathological progressions, such as cancer, inflammation, pathogenic infections, and autoimmune disease [8].

It is reported that CD146 is of great importance in tumor angiogenesis, metastasis, and chemoresistance [9]. For instance, CD146 has been demonstrated to mediate breast cancer cell invasion, epithelial mesenchymal transition (EMT), and metastasis [10]. In addition, previous studies confirmed that CD146 induced tamoxifen and cisplatin resistance in breast cancer cells and that its expression is associated with poor prognosis in breast cancer patients [11, 12]. However, the role of MCAM in CRC has rarely been reported.

Therefore, the comprehensive analysis of MCAM function in CRC and its TME was conducted. Our research reveals that MCAM, a novel biomarker, could reduce the proliferation, migration, and invasion ability of CRC cells and impact M1 macrophage polarization through regulating cytokines. This research may provide novel insights for regulating TME in the future.

## 2 Methods

### 2.1 Data Acquisition and Processing

The TIMER database (“http://timer.comp‐genomics.org/timer/”) was utilized to analyze the expression of MCAM across cancer datasets. The gene expression matrices and clinical characteristics of CRC patients were obtained from The Cancer Genome Atlas Program (TCGA, “https://www.cancer.gov/ccg/research/genome‐sequencing/tcga”), as well as from GSE28722, GSE14333, GSE103479, GSE39582, GSE71187, and GSE87211 cohort in the Gene‐Expression Omnibus (GEO, “https://www.ncbi.nlm.nih.gov/geo/”). All RNA‐seq data were standardized as log2 (TPM + 1) transformation using appropriate R packages before analysis.

### 2.2 Cell Culture and Cellular Transfection

The HCT116, HT29, and THP1 cells were obtained from the China Center for Type Culture Collection (Wuhan, China). HCT116 and HT29 cells were cultured in DMEM medium containing 10% fetal bovine serum (FBS) at 37°C in an incubator with 5% CO_2_. THP1 cells were cultured in RPMI‐1640 medium supplemented with 10% FBS under the same conditions. Lipo8000 Transfection Reagent (Beyotime Biotechnology, Shanghai, China) was used to transiently transfect cells plated in 6‐well plates with the MCAM overexpression plasmid and the empty plasmid purchased from MIAOLING BIOLOGY (Wuhan, China). Cells were used for subsequent experimental analysis at 48–72 h post‐transfection. The experiments were performed in triplicate.

### 2.3 qRT‐PCR (Real‐Time Fluorescence Quantitative PCR)

TRIzol (Invitrogen, USA) was used to extract cellular RNA, followed by qRT‐PCR as previously described [13]. The primers used were listed as follows: MCAM: F‐GGAAGCAGGAGATCACGCTA; R‐CCCATCTCTTCTGGGAGCTT; E‐cadherin: F‐AAGGTGCTCTTCCAGGAACC; R‐GCTGAGGATGGTGTAAGCGA; N‐cadherin: F‐CACCGTGGTCAAACCAATCG; R‐GAAGCCCTTCTTCTTGGCGA; MMP2: F‐GAGTGCATGAACCAACCAGC; R‐AGGTGTTCAGGTATTGCATGTG; MMP9: F‐TGACAGCGACAAGAAGTGGG; R‐TTCAGGGCGAGGACCATAGA; CD86: F‐CTGCTCATCTATACACGGTTACC; R‐GGAAACGTCGTACAGTTCTGTG; IL‐12b: F‐CAAACCTGACCCACCCAAGA; R‐AGGTGTCAGGGTACTCCCAG; TNF‐α: F‐CACAGTGAAGTGCTGGCAAC; R‐AGGAAGGCCTAAGGTCCACT; IL‐6: F‐TGCGCAGCTTTAAGGAGTTC; R‐CCCATGCTACATTTGCCGAA; IL‐23: F‐ACTTGTTGGGTGGCGTTAGA; R‐TCCCATCTCTGGTCCCCATT. The experiments were performed in triplicate.

### 2.4 Western Blot (WB) Assay and Immunohistochemistry (IHC)

WB and IHC were performed three times according to previously published protocols [13]. WB was conducted using specific antibodies, MCAM (1:2000, Proteintech, Wuhan, China, 17564‐1‐AP), GAPDH (1:7500, Proteintech, Wuhan, China, 60004‐1‐Ig), E‐cadherin (1:2000, Proteintech, Wuhan, China, 20874‐1‐AP), N‐cadherin (1:2000, Proteintech, Wuhan, China, 60335‐1‐Ig), MMP2 (1:1000, ABclonal, USA, A6247), and MMP9 (1:1000, ABclonal, USA, A0289). IHC was conducted with the specific antibodies, MCAM (1:200, Proteintech, Wuhan, China, 17564‐1‐AP), CXCL9 (1:200, Proteintech, Wuhan, China, 22355‐1‐AP), and CXCL10 (1:200, Proteintech, Wuhan, China, 10937‐1‐AP).

### 2.5 CCK8 (Cell Counting Kit 8) and Colony Formation Assay

HCT116 and HT29 cells were treated in 6‐well plates with MCAM overexpression plasmid and empty plasmid transfection. After 24 h of cell treatment, the cells in the 6‐well plates were counted, and 5000 cells were transferred to 96‐well plates. The relative cell viability was determined by measuring the absorbance at 450 nm at different time points using the CCK8 cell proliferation assay reagent (Wuhan, China). Thousand cells treated with plasmid transfection were transferred onto a 6‐well plate. After they have grown to an appropriate density, the cells were fixed with 4% paraformaldehyde for 20 min, and then the fixed colonies were stained with a crystal violet staining solution. Colonies were counted using ImageJ software. The experiments were performed in triplicate.

### 2.6 Wound Healing Assay

HCT116 and HT29 cells were transfected with an MCAM overexpression plasmid and an empty plasmid in a 6‐well plate until they reached 80% confluence. Subsequently, a straight line was drawn along the straight edge using a 10 μL white sterile tip gun. The cell scratch was evaluated by microphotography at 0 and 24 h post‐scratching. The scratched area was calculated by ImageJ software. The experiments were performed in triplicate.

### 2.7 Coculture System of CRC Cells and Macrophages

HCT116 and HT29 cells were plated separately in two 6 cm culture dishes. When the cell density reached about 70%–80%, plasmid transfection was conducted. After 48 h of culture, the supernatant of each group was collected into a sterile 5 mL centrifuge tube, centrifuged at 1000 rpm for 5 min, and then filtered through a 0.22um bacterial filter and transferred to a new 5 mL centrifuge tube. After centrifuging THP1 monocytes to remove the old medium, they were plated in a 6‐well plate at a concentration of 1 × 10^5^ cells/well, with a volume of 2 mL per well. Phorbol 12‐myristate 13‐acetate (PMA) was conducted at a concentration of 100 ng/mL to pretreat THP1 cells for 48 h. After the cells attached to the wall, they became macrophages. At this point, the old medium was discarded, and the aforementioned conditioned medium was treated with THP1 cells. After THP1 in the conditioned medium of CRC cells for 48 h of culture, the polarization status of THP1 was detected.

### 2.8 Development and Evaluation of a Nomogram

Univariate Cox (uni‐Cox) and multivariate Cox (multi‐Cox) regression analyses were conducted to evaluate the prognostic value of MCAM in CRC. The “rms” R package was used to establish a nomogram model and calibration curves. Receiver operating characteristic (ROC) curves for overall survival (OS) at 1, 3, and 5 years were generated using the “timeROC” R package. The area under the curve (AUC) values were calculated to assess the sensitivity and specificity of MCAM as a prognostic biomarker for CRC. Additionally, decision curve analysis (DCA) was performed using the “rmda” R package.

### 2.9 Differentially Expressed Genes (DEGs) Exploration

The TCGA‐COAD patients were divided into high and low MCAM expression groups based on the median MCAM expression level as the cutoff. The “limma” R package was adopted to calculate *p* value and log2 fold change (FC) of the whole genome genes between high MCAM and low MCAM patients. The significance criteria for identifying DEGs were set as adjusted *p* value < 0.05 and |log2 FC| > 1.29.

### 2.10 Gene Set Variation Analysis (GSVA)

The “GSVA” R package was used to explore differences in biological functions between high‐ and low‐MCAM groups.

### 2.11 Analysis of Correlation Between MCAM and M1 Macrophages

Initially, the “linkET” R package was utilized to explore the relationship between MCAM expression and common immune cells. Subsequently, the single‐sample gene‐set enrichment analysis (ssGSEA) algorithm was used to calculate ssGSEA scores for 28 different immune cell types. The infiltration levels of tumor‐infiltrating immune cells (TIICs) were computed using seven algorithms available in the TIMER database. Additionally, the correlation between MCAM and cytokines was analyzed and visualized using the “fmsb” R package.

### 2.12 Immunotherapy Efficacy, TIDE Scores, and MSI Status Analyses

The TIDE scores and MSI status of CRC samples were calculated using the Tumor Immune Dysfunction and Exclusion (TIDE, http://tide.dfci.harvard.edu/login/) database [14, 15]. Four cohorts (Gide et al. 2019, GSE91061, GSE78220, and GSE100797) treated with anti‐PD‐1, anti‐CTLA4 immunotherapy, and adoptive T cell therapy (ACT) were downloaded from the TIDE database (http://tide.dfci.harvard.edu/login/).

## 3 Statistical Analysis

All analyses were conducted using R software version 4.3.2. All experimental data were processed through GraphPad Prism 8.0 software. The Wilcoxon test or Kruskal–Wallis test was used to compare between two or multiple groups, respectively. The “survival” R package and logrank test were utilized to generate and analyze Kaplan–Meier (KM) survival curves, respectively. Statistical significance was defined as *p* < 0.05 for all comparisons.

## 4 Results

### 4.1 Low MCAM Expression Correlated With Poor Prognosis of CRC Patients

**FIGURE 1:**
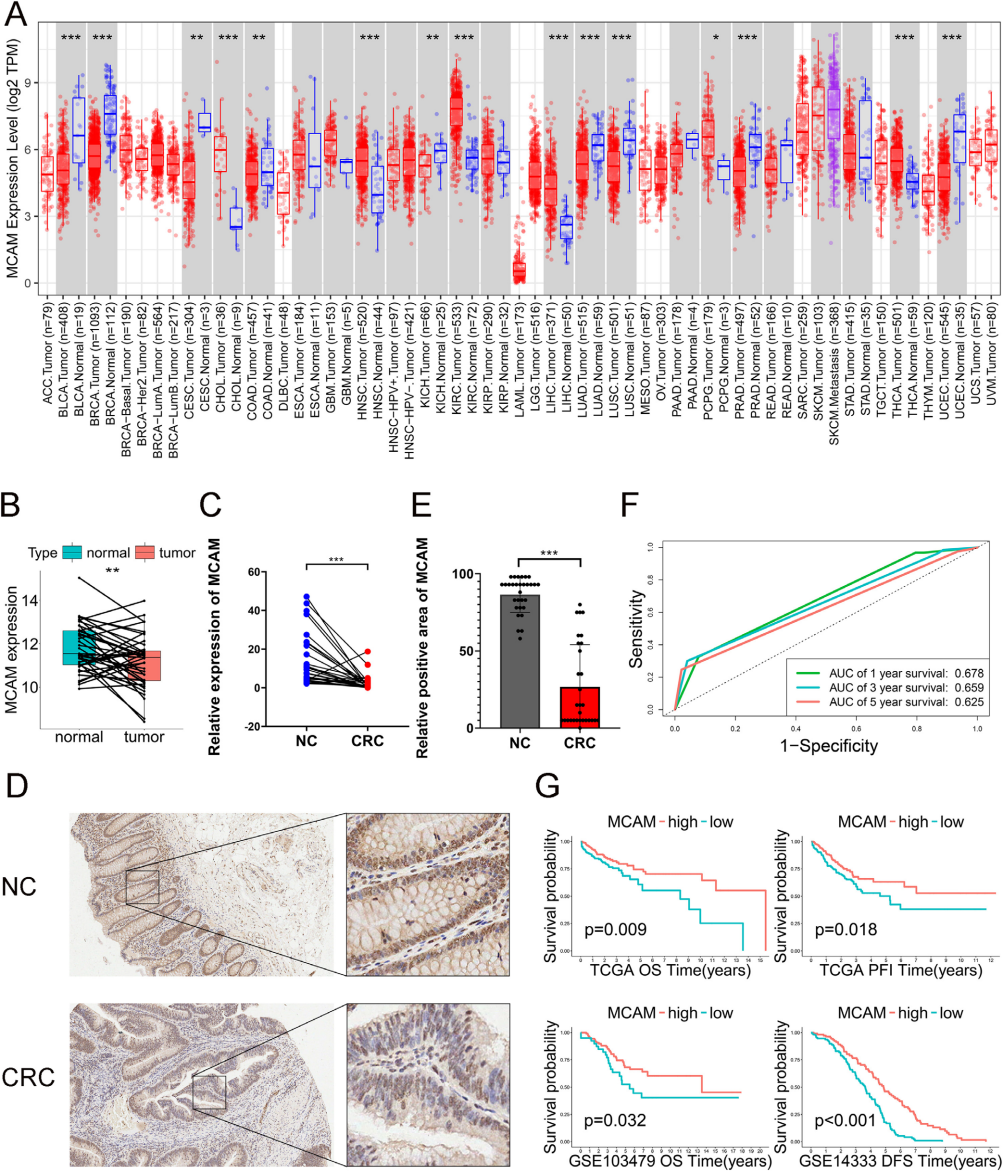
Expression and prognosis analysis about MCAM in the database. (A) MCAM expression in normal and tumor tissues in pan‐cancer data from the TIMER website. (B) MCAM expression in paired CRC samples from TCGA‐COAD. (C) qRT‐PCR analysis of paired CRC samples. (D, E) IHC analysis of adjacent noncancerous and CRC tissues. (F) ROC curve based on MCAM expression. (G) KM survival analysis of MCAM expression in TCGA‐COAD, GSE14333, and GSE103479 datasets showed patients expressing higher levels of MCAM had better prognosis. *p < 0.05; **p < 0.01; ***p < 0.001; CRC, colorectal cancer; qRT‐PCR, Real‐time fluorescence quantitative PCR; IHC, immunohistochemistry.

In pan‐cancer analysis, MCAM gene expression was significantly lower in various common cancers compared to normal tissue, including colon, lung, and prostate (Figure 1A). Further studies on MCAM expression revealed a similar reduction in CRC tissues compared to adjacent noncancerous tissues (Figure 1B. Figure S1A–C). In addition, qRT‐PCR and IHC analysis further demonstrated the downregulation of MCAM at transcriptional and translational levels in CRC (Figure 1C–E). Furthermore, time‐dependent ROC curves for assessing 1‐, 3‐, and 5‐year OS based on MCAM expression levels yielded AUC values of 0.678, 0.659, and 0.625, respectively (Figure 1F). Additionally, KM curves indicated that low MCAM expression was associated with poor prognosis for CRC patients (Figure 1G). These findings suggest that MCAM plays a protective role in CRC.

### 4.2 MCAM Expression Correlates With Clinicopathological Factors and the Construction of Nomogram Model

**FIGURE 2:**
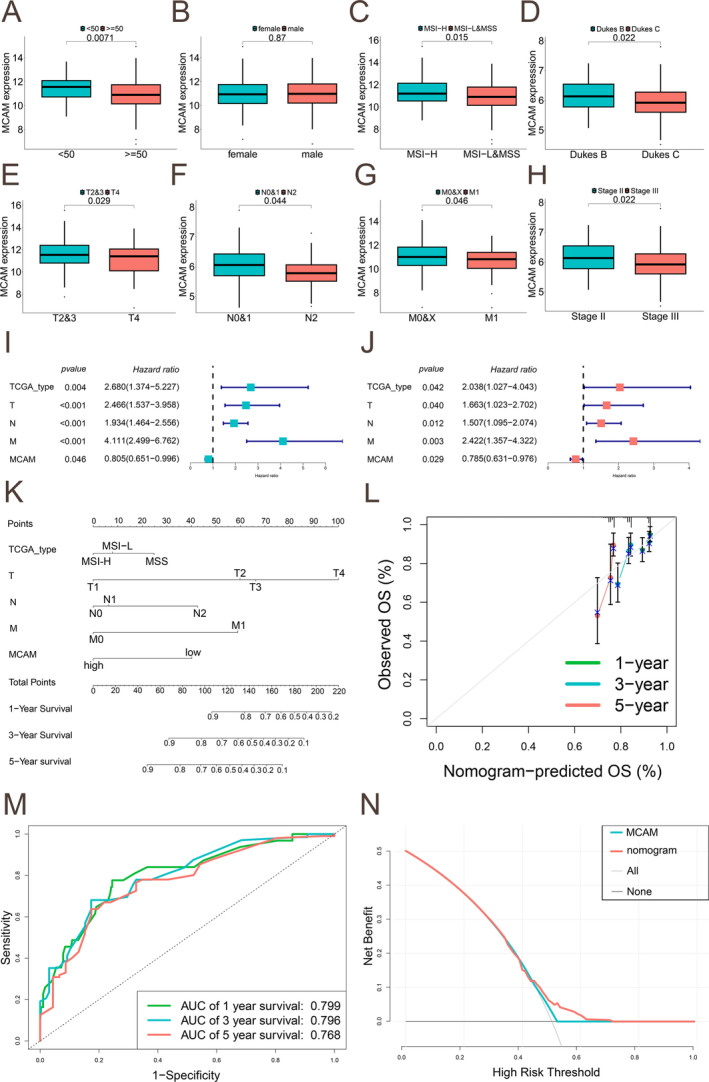
MCAM expression correlates with patient clinicopathological parameters and nomogram construction. (A) Differential analysis for MCAM expression in patients of different ages. (B) gender. (C) TCGA type. (D) Dukes stage. (E) T stage. (F) N stage. (G) M stage. (H) pathological stage. (I, J) Uni‐Cox and multi‐Cox regression analyses for clinical factors and MCAM expression with OS. Hazard ratio > 1 represented risk factors for survival, and hazard ratio < 1 represented protective factors. (K) Nomogram for predicting the 1‐, 3‐, and 5‐year OS from TCGA‐COAD. (L) Calibration curves for predicting 1‐, 3‐, and 5‐year OS. (M) ROC curve for predicting 1‐, 3‐, and 5‐year OS. (N) DCA for determining survival. OS, overall survival; CRC, colorectal cancer; uni‐Cox, univariate Cox; multi‐Cox, multivariate Cox; ROC, receiver operating characteristic; DCA, decision curve analysis.

The correlation between MCAM expression and clinicopathological characteristics of TCGA‐COAD patients was analyzed, revealing lower MCAM expression in advanced T/N/M stage, pathological stages, and Dukes stages (Figure 2A–H). To further validate the predictive capacity of MCAM for the prognosis of CRC patients, uni‐Cox and multi‐Cox regression analyses were performed to explore the relationship between MCAM expression and OS in CRC patients. As shown in the uni‐Cox regression analysis, TCGA_type (HR = 2.68, *p* = 0.004), T stage (HR = 2.466, *p* < 0.001), N stage (HR = 1.934, *p* < 0.001), and M stage (HR = 4.111, *p* < 0.001) were negatively correlated with OS, whereas MCAM (HR = 0.805, *p* = 0.046) was positively correlated with OS (Figure 2I). Additionally, multi‐Cox regression analysis indicated that MCAM served as an independent prognostic factor for OS in CRC (Figure 2J). Subsequent analysis demonstrated that MCAM could accurately predict the 1, 3, and 5‐year patient prognosis by constructing a nomogram model (Figure 2K). The reliability of this model was validated through calibration curves (Figure 2L). Furthermore, the AUC values of 0.799, 0.796, and 0.768, respectively, indicating a high prediction accuracy (Figure 2M). Finally, DCA illustrated that the clinical utility of this model is better (Figure 2N).

### 4.3 MCAM Inhibited CRC Cell Proliferation, Migration, and Invasion

**FIGURE 3:**
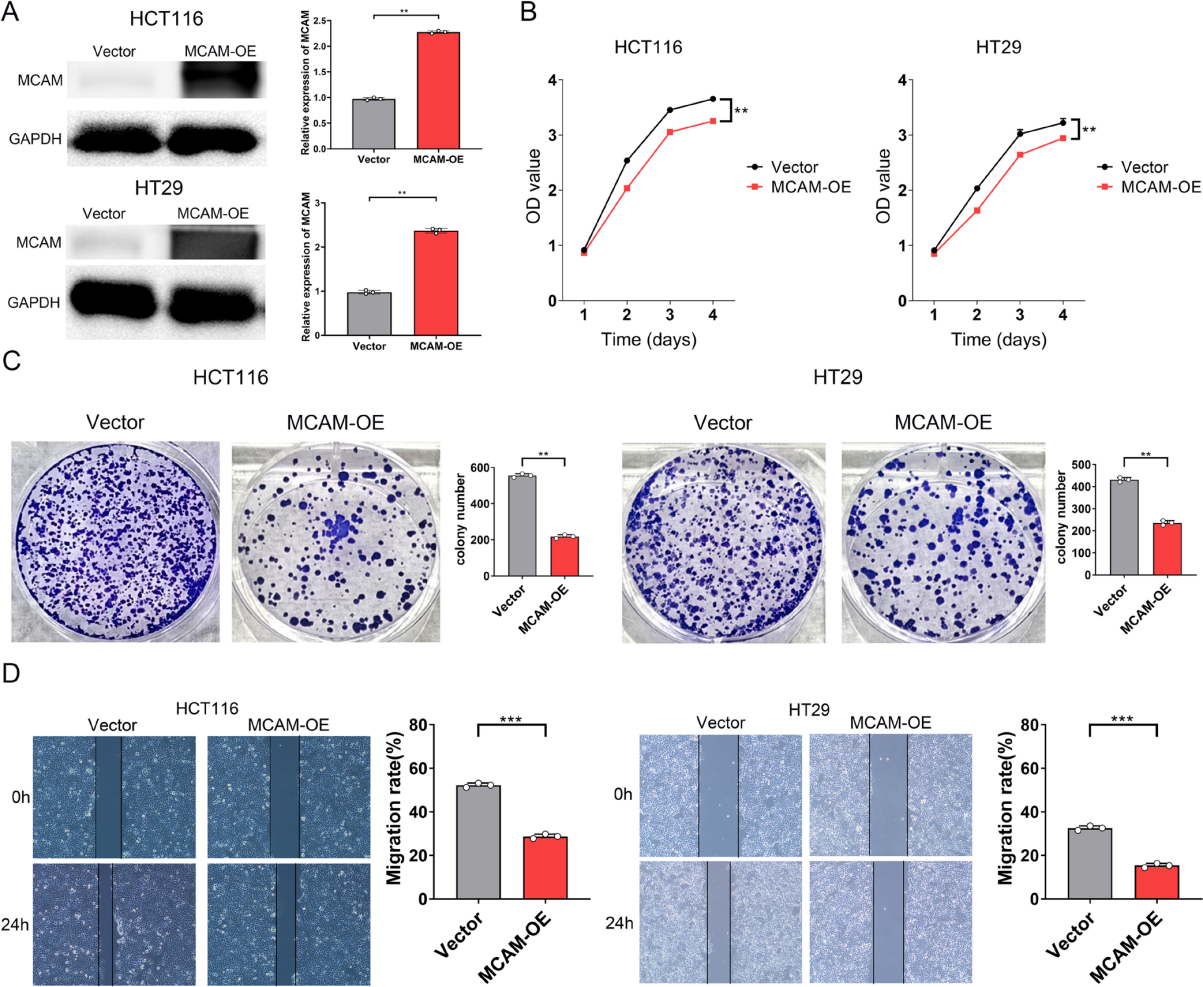
MCAM inhibits proliferation, migration, and invasion of CRC cells. (A) Evaluation of MCAM overexpression at mRNA and protein levels. (B) CCK8 assay showed that MCAM overexpression inhibited proliferation. (C) Colony formation assay revealed that MCAM reduced colonies. (D) MCAM inhibited invasion in the wound healing assay. CCK8, Cell Counting Kit 8. **p < 0.01, ***p < 0.001.

To evaluate the transfection efficiency in HCT116 and HT29 cells, we performed qRT‐PCR and WB analysis, showing that MCAM gene expression was significantly increased at transcriptional and translational levels in HCT116 and HT29 cells (Figure 3A). Next, CCK‐8 assays showed that MCAM overexpression was sufficient to inhibit CRC cell viability (Figure 3B). In addition, cloning assays demonstrated consistent results, where MCAM overexpression impaired the proliferation ability of CRC cells (Figure 3C). Finally, wound healing assays indicated that upregulated MCAM significantly inhibited CRC cell migration and invasion compared to the control group (Figure 3D). In summary, MCAM overexpression suppresses the proliferation, migration, and invasion of CRC cells.

### 4.4 Functional Enrichment Analysis of MCAM in TCGA‐COAD Patients

**FIGURE 4:**
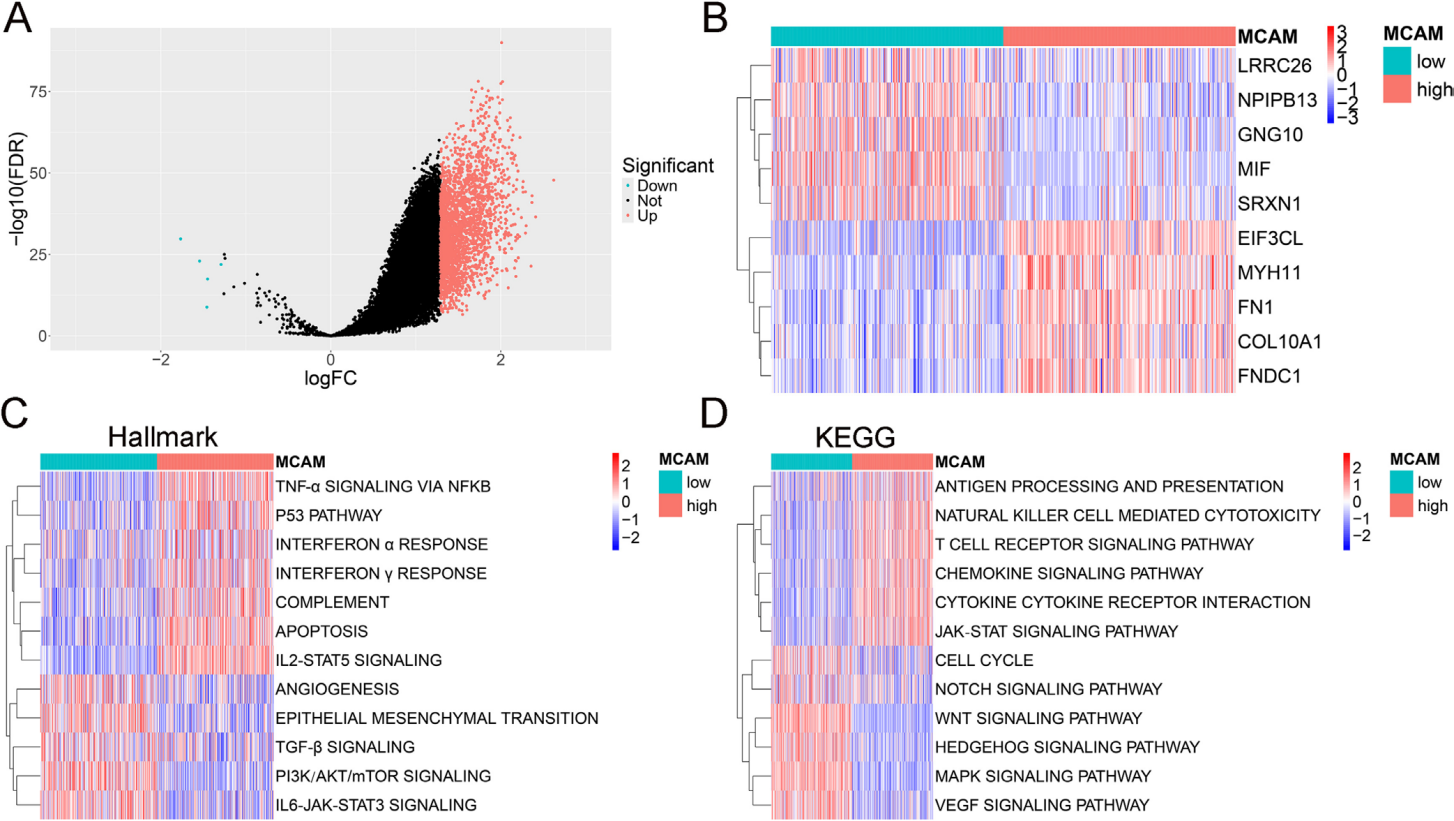
MCAM‐related functional enrichment analysis in CRC. (A) Volcano plot from DEGs between high‐ and low‐MCAM groups. (B) Heat map shows the top five genes in both upregulated and downregulated DEGs. (C, D) GSVA shows the activation status of biological pathways in the two groups using Hallmark and KEGG gene sets. DEGs, differentially expressed genes; GSVA, gene set variation analysis.

The upregulated and downregulated DEGs were clearly distinguished through differential analysis between high MCAM and low MCAM groups (Figure 4A; Table S1). Furthermore, upregulated genes including EIF3CL, MYH11, FN1, COL10A1, and FNDC1 were identified in the high MCAM group compared to the low MCAM group, as well as downregulated genes including LRRC26, NPIPB13, GNG10, MIF, and SRXN1 (Figure 4B). Further, DEGs were selected for GSVA to explore potential functional pathways in CRC. Hallmark gene sets analysis showed that high MCAM was closely related to IL2‐STAT5 [16], TNF‐α [17], and other immune‐related signaling pathways (Figure 4C; Table S2). Similarly, KEGG analysis showed that low MCAM was mainly related to Wnt [18], MAPK [19], and other signaling pathways involved in immune evasion (Figure 4D; Table S3). These results indicate that elevated MCAM expression is linked to numerous immune pathways in CRC.

### 4.5 MCAM Reduced CRC Metastasis by Inhibiting N‐Cadherin and MMP‐2/9 Expression

**FIGURE 5:**
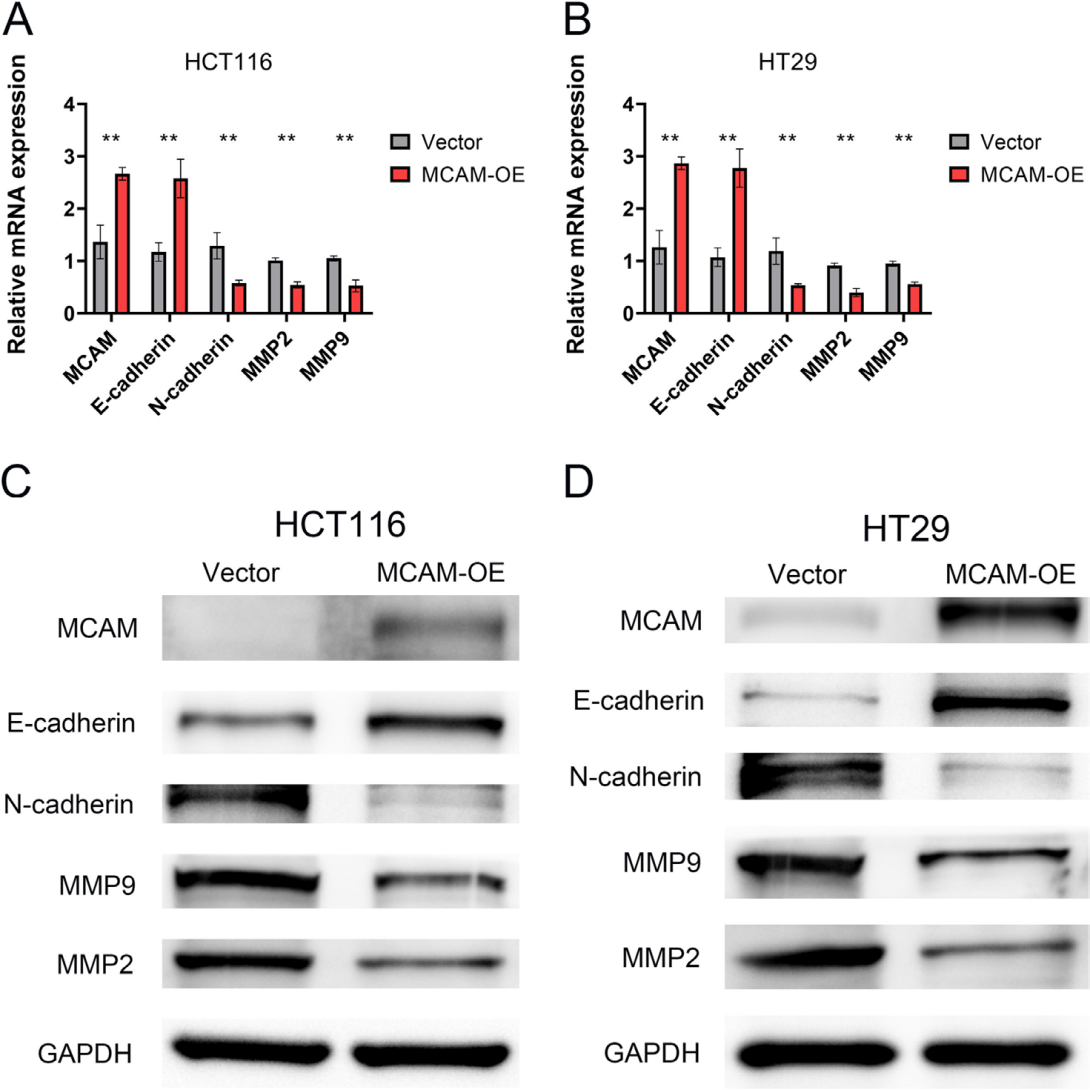
The impact of MCAM on EMT phenotype and MMP2/9 expression. (A, B) MCAM increased the expression of E‐cadherin and decreased the expression of N‐cadherin, MMP2, and MMP9 at mRNA levels. (C, D) MCAM increased the expression of E‐cadherin and decreased the expression of N‐cadherin, MMP2, and MMP9 at protein levels. EMT, epithelial mesenchymal transition. **p < 0.01.

The aforementioned results demonstrated that MCAM inhibited CRC proliferation, metastasis, and exhibited a negative correlation with EMT phenotype. To further elucidate the molecular mechanisms underlying MCAM‐mediated metastasis inhibition, we detected the expression levels of EMT markers and MMP‐2/9, which were known to play pivotal roles in CRC metastasis and invasion [20, 21]. As shown in Figure 5A,B, MCAM overexpression decreased the mesenchymal marker (N‐cadherin) and MMP‐2/9 expression, while increasing the epithelial marker (E‐cadherin) expression at transcriptional levels. Additionally, similar results were observed at translational levels (Figure 5C,D). In sum, these data demonstrated a mechanism by which MCAM reduces metastasis by inhibiting EMT in CRC cells.

### 4.6 MCAM Influences TME and Regulates Macrophage Polarization

**FIGURE 6:**
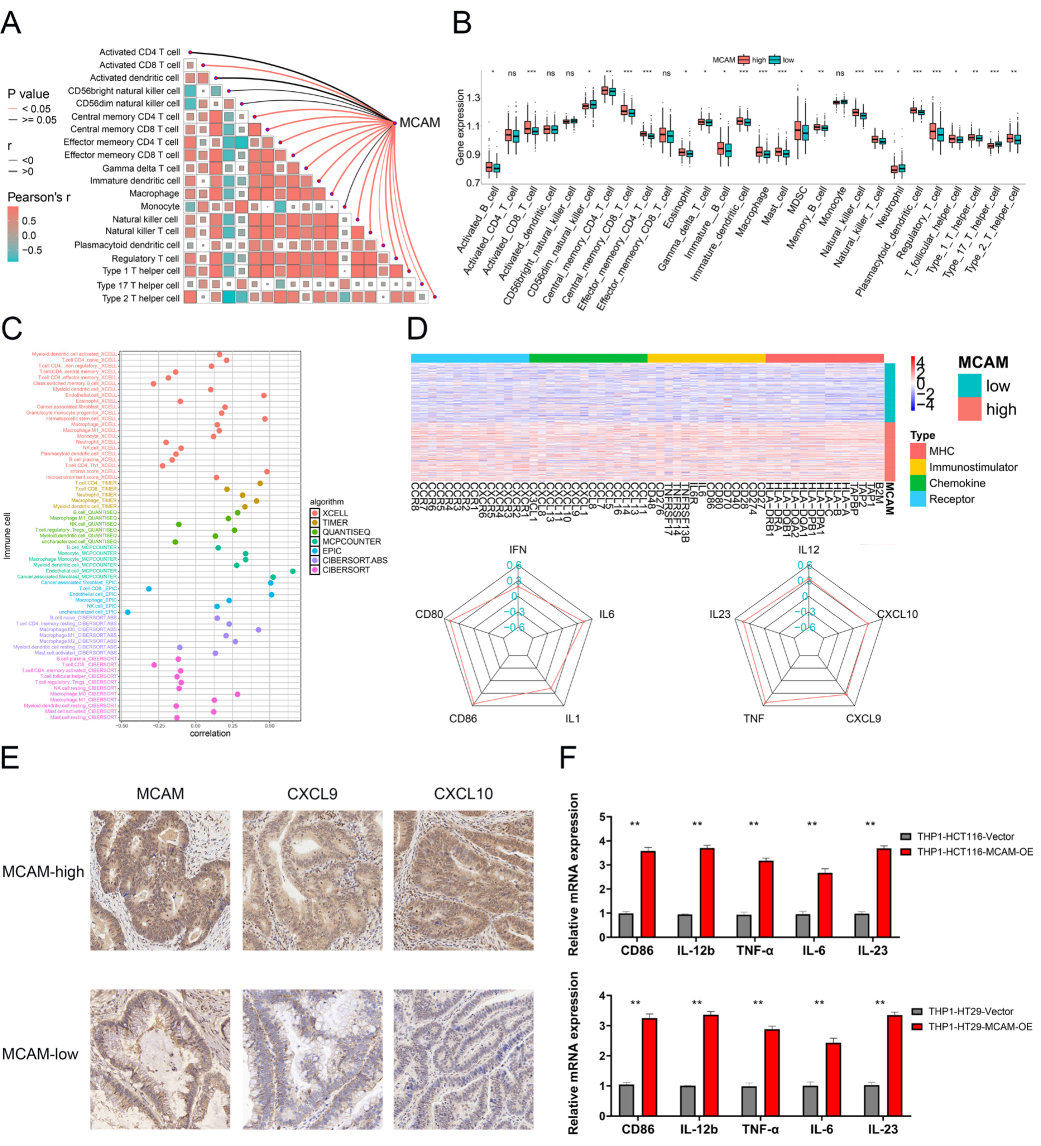
Relationship for MCAM with M1 macrophages. (A) Correlation between MCAM expression and common immune cells. (B) The varied proportions of 28 TIICs in two groups. (C) Correlation between MCAM expression and infiltration levels of TIICs calculated by seven independent algorithms. (D) Correlation between MCAM and cytokines. (E) MCAM increased CXCL9/10 levels in IHC analysis. (F) MCAM increased the expression of cytokines secreted by M1 macrophages. *p < 0.05, **p < 0.01, ***p < 0.001, ns (*p* > 0.05); IHC, immunohistochemistry; TIICs, tumor‐infiltrating immune cells.

Functional enrichment analysis indicated that MCAM was associated with immune; we further investigated the impact of MCAM on immune. Initially, high MCAM was found to be positively linked to more immune cells, including macrophages (Figure 6A), and also boosted macrophage infiltration (Figure 6B). Further analysis revealed a positive correlation between MCAM expression and immune cells, especially increased infiltration of M1 macrophages, as calculated by seven algorithms (Figure 6C). Studies have reported that M1 macrophages, upon stimulation by bacterial products like lipopolysaccharide (LPS), can be induced by IFN‐γ to secrete high levels of pro‐inflammatory cytokines, such as TNF‐α, IL‐6, IL‐12, IL‐23, CXCL9, and CXCL10 [22, 23]. To further explore the molecular mechanisms underlying MCAM overexpression‐induced M1 macrophage infiltration in CRC, we analyzed the correlation between MCAM and M1 macrophage‐related molecules. Notably, CD80/86, a marker of M1 macrophages, and other chemokines exhibited a significantly positive correlation with high MCAM expression, as shown in Figure 6D. The above results were validated by external datasets (Figure S2A–D). Furthermore, IHC analysis further revealed that high MCAM led to upregulated expression of CXCL9/10 (molecules secreted by M1 macrophages) (Figure 6E). Finally, qRT‐PCR analysis showed that MCAM overexpression in CRC cells was sufficient to increase the expression of CD86, IL12b, and other important molecules in THP1 cells (Figure 6F). In summary, MCAM may promote M1 macrophage polarization by enhancing the expression of some key molecules in TME.

### 4.7 High MCAM Expression Correlated With Better Immunotherapy Efficacy

**FIGURE 7:**
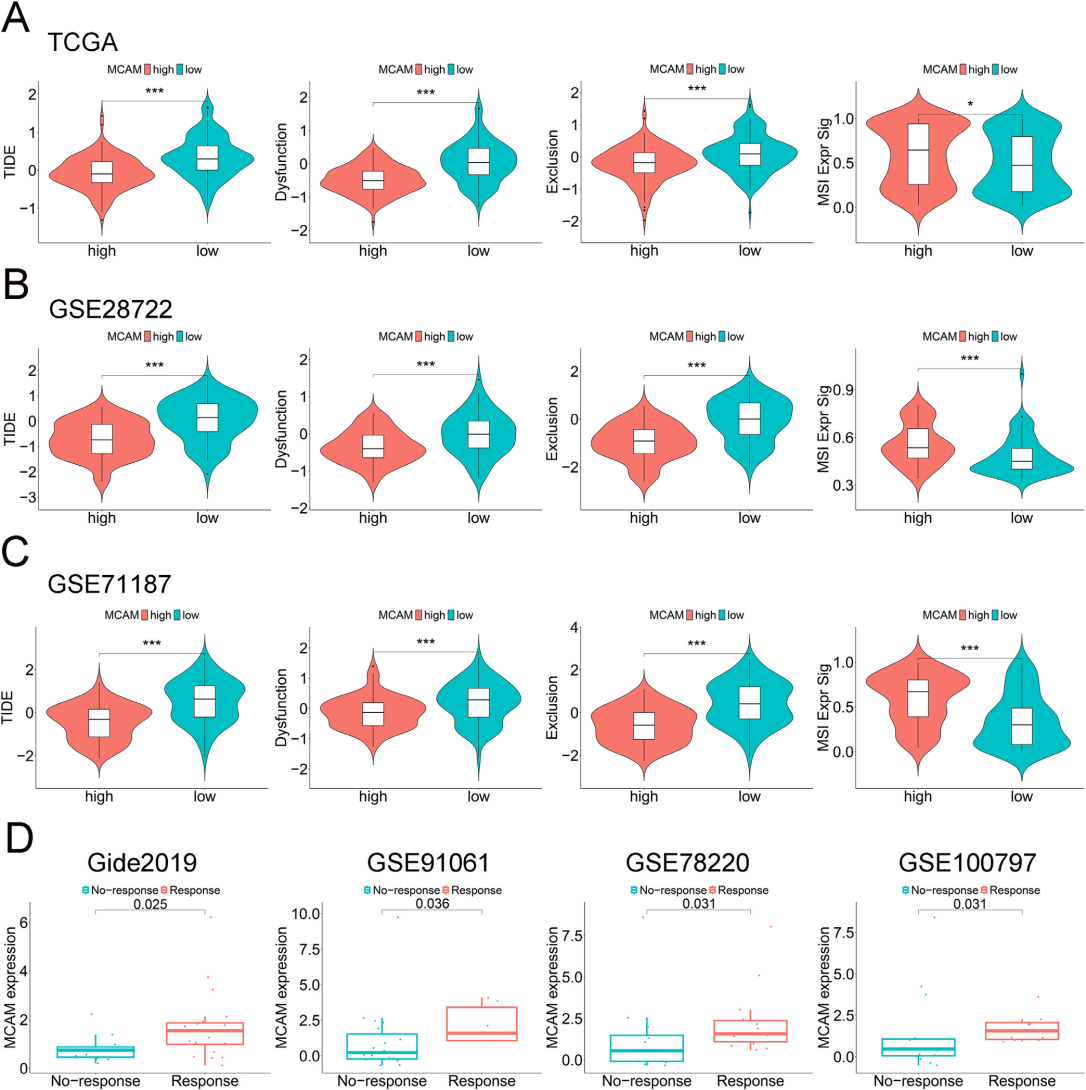
Immunotherapy efficacy analysis and TIDE prediction. (A) Correlation of MCAM expression with TIDE, dysfunction, exclusion, and MSI in TCGA. (B) GSE28722. (C) GSE71187. (D) Patients with high levels of MCAM exhibited a better response to immunotherapy.  *p < 0.05, ***p ≤ 0.001.

To investigate the impact of elevated MCAM expression on immunotherapy efficacy in CRC patients, we employed TIDE to evaluate the potential immunotherapeutic efficacy in two groups. A higher TIDE predictive score indicates a stronger propensity for immune evasion, suggesting a lower possibility of patients benefiting from immune checkpoint inhibitor (ICI) therapy [14]. Microsatellite instability (MSI) has been demonstrated to be closely linked with long‐term immunotherapy responses and improved prognosis in CRC patients treated with ICI [24]. High MCAM expression was associated with lower TIDE scores, Dysfunction scores, Exclusion scores, and higher MSI scores from the TCGA dataset (Figure 7A). Furthermore, consistent results were observed in the GSE28722 and GSE71187 datasets (Figure 7B,C). Finally, the correlation analysis between MCAM expression and immunotherapy efficacy revealed that patients with high MCAM levels led to better responses to ICI treatment (Figure 7D). In summary, these findings suggest that elevated MCAM expression may improve immunotherapy efficacy in CRC patients.

## 5 Discussion

In recent years, some researchers have reported that MCAM‐mediated cell invasion, EMT, and metastasis in breast cancer [10], while CD146 depletion significantly inhibited T‐cell antitumor response in tumor models [25]. However, only a few researchers have simply revealed the role of MCAM in CRC [26], and comprehensive analysis of MCAM in CRC remains largely unexplored, sparking our eager curiosity. In this study, we found that high MCAM expression indicated better prognosis, higher infiltration levels of M1 macrophages, and better immunotherapy efficacy, which suggested that MCAM plays a pivotal role in immune activation.

In this study, we discovered that high levels of MCAM reduced the proliferation, migration, and invasion abilities of CRC cells. EMT occupies a central role in the processes of tumorigenesis and metastasis [27, 28]. During the EMT process, epithelial tumor cells undergo notable morphological and phenotypic transformations. These changes include the loss of tight junctions, alterations in cell polarity, and reorganization of the cytoskeleton, which confer cells with increased invasiveness and phenotypic changes [29]. To delve deeper into the molecular mechanism underlying CRC cells metastasis, a preliminary analysis of the EMT phenotype was conducted, revealing that MCAM overexpression decreased N‐cadherin and MMP‐2/9 expression while increasing E‐cadherin expression in CRC cells. TGF‐β1 has been identified as the most potent factor capable of independently inducing and activating EMT in a multiple types of cancer cells [30, 31, 32]. Previous studies have demonstrated that CD146 affected EMT in ovarian cancer through TGF‐β signaling [33]. In addition, TGF‐β signaling was found to be enriched in the low‐MCAM group. Therefore, we reasonably infer that MCAM functions as an inhibitor of EMT, inhibiting TGF‐β signaling.

We elucidated the association between MCAM and M1 macrophages through TME analysis. Previous studies demonstrate a positive correlation between CD146 and M1 macrophage infiltration in clear cell renal cell carcinoma (ccRCC) [34]. Consistent with this research, our investigation yielded the same results. The M1‐like polarization of macrophages is critical for tumor suppression [35]. Through cytokines or chemokines (such as IL6, IL12b, and TNF‐α), it can inhibit the recruitment of myeloid cells while activating antitumor and immune‐killing functions. One of the most promising strategies in cancer treatment involves the application of M1 macrophages for antitumor delivery [36, 37]. Recent studies have reported that high CD146 expression in macrophages promoted M1 polarization and enhanced antitumor immunity by activating the NLRP3 inflammasome in hepatocellular carcinoma (HCC) [38], and depletion of MCAM in mammary basal cells promoted the recruitment and activation of M2 macrophages via the IL4‐STAT6 axis, thereby promoting mammary epithelial cell proliferation [39]. In our study, the upregulated expression of CD86, TNF‐α, and IL12b in THP1 cells coculturing with CRC cells was induced by MCAM overexpression in CRC cells. Therefore, we consider that MCAM may promote M1 macrophage polarization by increasing cytokine levels. It is worthwhile to utilize flow cytometry and establish animal models to further validate the above results and explore the molecular mechanism regulating M1 macrophage polarization.

The battle against tumors has long been a central stage in both clinical and experimental research. In recent years, immunotherapy emerged as a breakthrough strategy in cancer treatment, offering innovative and promising strategies for cancer treatment. In this study, patients exhibiting high levels of MCAM demonstrated improved responses to common immunotherapies including anti‐PD‐1, anti‐CTLA4, and ACT. Notably, biomarkers such as TIDE score have been reported to predict patient response to immunotherapy [40]. Subsequently, we further explore the potential immunotherapeutic efficacy in CRC patients, revealing that high TIDE scores were associated with low levels of MCAM. The TIDE prediction score is associated with T‐cell dysfunction in high‐CTL tumors and T‐cell exclusion in low‐CTL tumors, thereby representing two distinct immune escape mechanisms [40]. Therefore, low levels of MCAM led to immune evasion in CRC, which was consistent with the aforementioned results obtained through GSVA. Research has shown that the TIDE score can predict patient prognosis receiving first‐line anti‐PD1 or anti‐CTLA4 antibodies with greater accuracy than other biomarkers, including PD‐L1 levels and mutation burden [40]. In summary, MCAM enhances benefits to immunotherapy for CRC patients.

Recent studies have shown that MSI significantly impacts the immunotherapy efficacy [41, 42]. MSI is commonly found in CRC [24], and the high mutational burden resulting from MSI renders tumors immunogenic and sensitive to anti‐PD1 therapy [43, 44]. In this study, it was found that high MCAM expression is linked to high MSI scores, indicating better immunotherapy efficacy. Additionally, it was associated with MSI‐H patients, leading to the accumulation of tumor‐associated gene mutations and the neoantigen generation, which enhances antitumor immune response [41]. Overall, we propose that MCAM plays a role in influencing the immunotherapy efficacy in CRC patients, potentially by modulating MSI status.

While the findings of this study largely aligned with our expectations, there were still several notable limitations to consider. Flow cytometry and animal models were not established to further demonstrate the impact of MCAM overexpression in CRC cells on M1 macrophage polarization. In addition, the molecular mechanism of MCAM regulating the EMT phenotype was also not explored.

In conclusion, this study demonstrates that upregulated MCAM expression is linked to a better prognosis and decreased proliferation, migration, and invasion abilities in CRC. Additionally, MCAM overexpression in CRC cells impacts M1 macrophage polarization in the TME. Furthermore, high MCAM expression induces immune activation, which supports the increased responsiveness of CRC to immunotherapy. These findings underscore the significant clinical significance of MCAM and offer new perspectives into immunotherapy for CRC.

## Author Contributions

All authors contributed to the study conception and design. Jingkai Zhou collected all public datasets and was responsible for the main analysis, then wrote this original draft. Haihang Nie and Jing Liu completed IHC, qRT‐PCR, and WB. Yali Yu reviewed and edited this original draft. Chao Yang and Yuntian Hong were responsible for prognosis, TME analysis, and data integration. Haizhou Wang was responsible for the collection of CRC tissues. Yumei Ning and Jun Lai conducted the construction of cell models, CCK‐8 assay, colony formation assay, and wound healing assay. Fan Wang and Xuelian Tang were responsible for funding acquisition and supervision of the study. Qiu Zhao was the lead author of the study and guided the selection of analysis. All authors read and approved the final manuscript.

## Ethics Statement

The study was approved by the Research Ethics Committee of Zhongnan Hospital of Wuhan University, and written informed consent was obtained from all patients (grant no. 2024049). All the methods were carried out in accordance with the relevant guidelines under the ethical approval and consent to participate section.

## Consent

All authors agree to this manuscript's publication.

## Conflicts of Interest

The authors declare no conflicts of interest.

## Supporting information


Data S1.

Data S2.

## Data Availability

All public datasets enrolled in this study could be downloaded from the GEO database (https://www.ncbi.nlm.nih.gov/geo/) and the TCGA database (https://www.cancer.gov/ccg/research/genome‐sequencing/tcga). All data generated or analyzed during this study are included in the Supporting Information files of this article.
