# Supplementary material for: Melanoma Cell Adhesion Molecule Plays a Pivotal Role in Proliferation, Migration, Tumor Immune Microenvironment, and Immunotherapy in Colorectal Cancer
Source: Cancer Med. 2025 Mar 5;14(5):e70740. doi: 10.1002/cam4.70740 (PMC11880918; doi:10.1002/cam4.70740)
Supplement: Supplementary file 1 — Data S1. [file CAM4-14-e70740-s002.docx]

**Supplementary Figures**


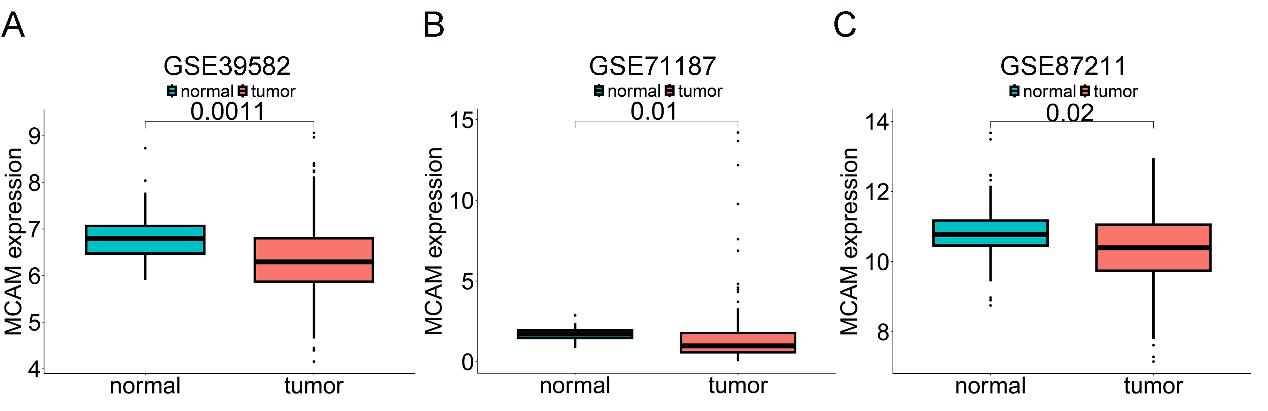


**Figure S1** The expression of MCAM in CRC. **A-C** Differential expression of MCAM between normal and tumor samples from GSE39582, GSE71187, and GSE87211. CRC, colorectal cancer.


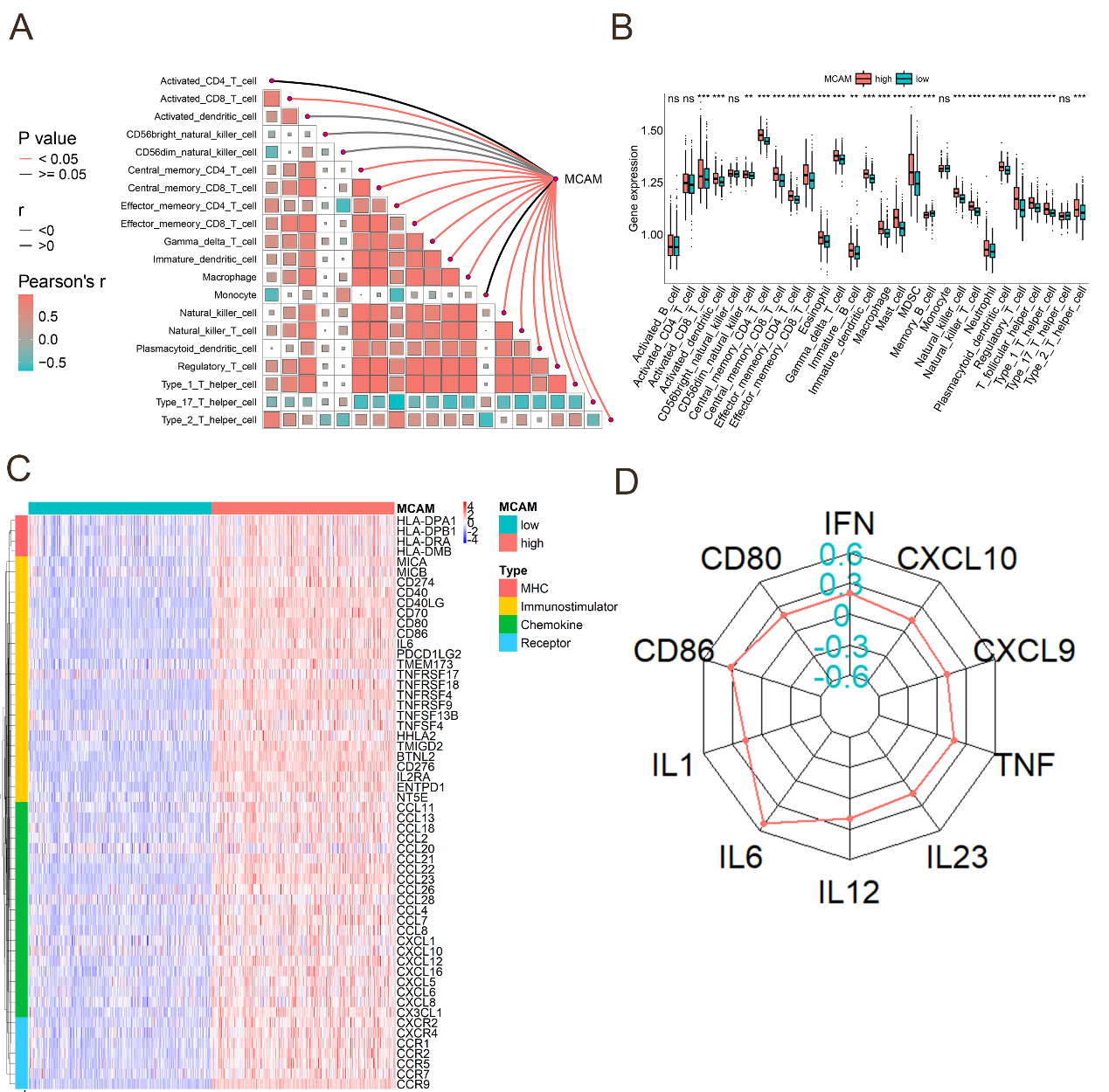


**Figure S2** The impact of MCAM on M1 macrophages in GEO database. **A** Correlation between MCAM expression and common immune cells. **B** The varied proportions of 28 TIICs in two groups. **C** Expression levels of 4 immunomodulators (MHC, immunostimulators, chemokines and receptors) in high and low-MCAM groups. **D** Correlation between MCAM and cytokines secreted by M1 macrophages. *p<0.05, **p<0.01, ***p<0.001, ns (p>0.05); TIICs, tumor-infiltrating immune cells.
